# Salidroside: A Potential Drug Candidate to Treat Rheumatoid Arthritis

**DOI:** 10.3390/molecules30193865

**Published:** 2025-09-24

**Authors:** Jiaying Guo, Shan Jiang, Mei Liu, Min Wang, Beibei Han, Ning Zhang, Yumei Liao, Yinhong Xiang, Jianxin Liu, Huifeng Sun

**Affiliations:** 1 School of Pharmacy, Heilongjiang University of Chinese Medicine, Harbin 150040, China; jiayingguo123@126.com (J.G.); 18145413520@163.com (S.J.); wangminmin0123@126.com (M.W.); han_04133@163.com (B.H.); zhangning0454@163.com (N.Z.); 2 Sino-Pakistan Center on Traditional Chinese Medicine, School of Pharmaceutical Sciences, School of Basic Medical Sciences, China-Pakistan International Science and Technology Innovation Cooperation Base for Ethnic Medicine Development in Hunan Province, Hunan University of Medicine, Huaihua 418000, China; lm1003573291@126.com (M.L.); umi11142013@foxmail.com (Y.L.); xiangyh@hnmu.edu.cn (Y.X.)

**Keywords:** salidroside (SAL), rheumatoid arthritis (RA), pharmacological effects, anti-inflammatory, immune system

## Abstract

Rheumatoid arthritis (RA) is a widespread autoimmune disease that significantly impacts the lives of RA patients. It is often typified as swelling and deformation of small joints, as well as systemic inflammation. *Rhodiola rosea* has been utilized for millennia to treat various ailments and is known to contain numerous active compounds, including saponins, volatile oils, coumarins, and flavonoids. Recent studies have underscored the pivotal role of salidroside (SAL), a key constituent of *Rhodiola rosea* L. Modern research indicates that SAL has various pharmacological activities, such as its antioxidant, anti-inflammatory, anti-fatigue, and anti-cancer effects. Despite this, the pathogenesis of RA remains highly complex, and a notable lack exists in overview studies investigating the anti-RA mechanisms of SAL. Therefore, the purpose of this article is to review the present research efforts on the anti-RA mechanisms of SAL and to explore future research prospects for this compound.

## 1. Introduction

RA is a long-term inflammatory disease of the joints that leads to cartilage and bone damage as well as disability [1]. Being a systemic autoimmune illness, RA commonly impacts other tissues and organs as well as the joints, including the heart, kidneys, gastrointestinal tract, nerves, and so on [2].

Therefore, a timely early evaluation of RA is crucial to avoid serious arthritic injuries such as physical disability. Autoantibodies as biomarkers can help practitioners make an efficient diagnosis and achieve the desired therapeutic outcome. Effective treatments for RA include oral administration of traditional synthetic modified DMARDs (smDMARDs), including methotrexate, targeted synthesis DMARDs (ts DMARDs) such as Janus kinase (JAK) inhibitors or tumor necrosis factor inhibitor (TNFi) biologics, and the recently discovered proteasome inhibitors (PIs) [3,4,5,6]. Although early and aggressive use of pharmacologically palliative antirheumatic drugs can significantly improve clinical and radiological outcomes, some patients have been reported to have significant problems with emotional and social functioning that reflect the unmet needs of RA patients [7]. Therefore, non-pharmacological treatments for RA patients are also a means of relieving the symptoms of arthritis. Patients with RA can benefit from dietary intervention with the Mediterranean diet as a complement and extension of drug therapy [8]. However, in relation to the disease itself, appropriate exercise and diet can only be considered a supplementary treatment. Tapping into an efficient and therapeutically effective anti-RA drug is essential for the treatment of RA. SAL is compared with the current RA treatment drugs, as shown in Table 1.

*Rhodiola rosea*, a worldwide adaptogen, protects against inflammation in diseases like heart disease, neurodegenerative diseases, and cancer [9]. SAL is a tyrosol glucoside isolated from the roots of *Rhodiola rosea* Spp. [10]. It has attracted the attention of researchers because of its many biochemical and pharmacological features. During the latest decade, the medicinal properties of SAL have been gradually discovered in popular studies, such as anti-cancer, antioxidant, anti-inflammatory, and immunomodulatory agents [11]. SAL also promotes blood vessel growth, protects bone cells against glucocorticoids, and prevents steroid-induced osteonecrosis. It also fights inflammation by blocking TNF-α and IL-6 while encouraging IL-10 [12]. SAL has a pharmacological effect on the regulation of glial cell polarization and the reduction in neuroinflammation [13]. Based on the pharmacological effects of SAL and the current urgent need for a compound to treat RA, we summarized the induction mechanism of RA, the latest research results of SAL against RA, and prospects for SAL in anti-RA.

## 2. Mechanisms of RA

**Figure 1:**
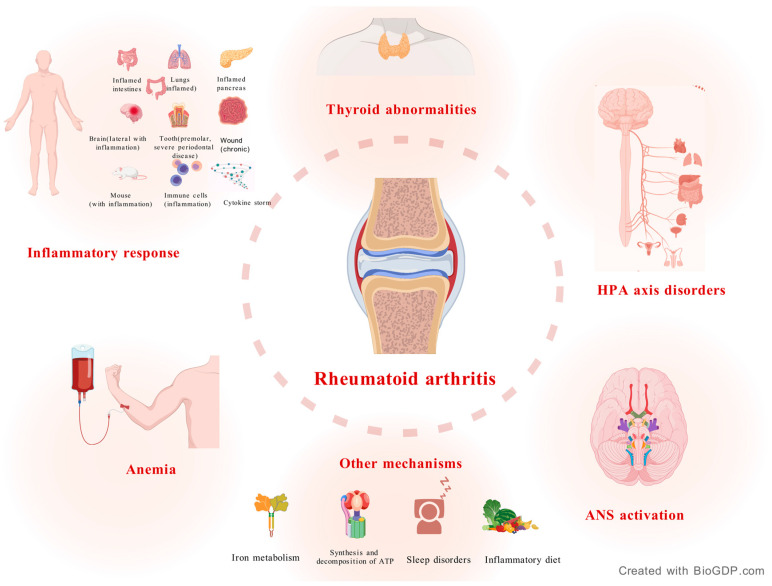
Pathogenesis of RA. RA is strongly associated with an inflammatory response. HPA axis disorders, autonomic nervous system activation, anemia, and thyroid metabolic abnormalities are also major manifestations of RA. In addition, late nights, iron imbalance, and diet can also affect RA.

As an autoimmune disease, RA is characterized by chronic and systemic inflammation and has been linked to various complications in the cardiovascular and respiratory systems [14]. Rheumatoid arthritis is a multi-stage and complex process involving multiple aspects such as genetic, environmental, and behavioral risk factors. These factors may lead to damage to the immune system and induce autoimmune processes like autoantibody production and inflammation. Although diagnosed RA usually involves joints, the key to the pathogenesis of the disease may not be present in the joints: association with citrulline production, the gut microbiome, or adipose tissue in lung or periodontal tissues supports the normal findings in synovial tissues of patients with arthralgia. Anti-citrullinated protein antibody (ACPA) [15,16,17,18]. Years before the onset of RA, the presence of ACPA can be detected. Possible mechanisms involved in RA are summarized in Table 2 and Figure 1.

### 2.1. Inflammatory Response

RA is a chronic autoimmune disease [31]. Autoimmunity in RA may initially occur at mucosal sites. Serological and other tests may first detect autoimmunity in the blood, which later develops into inflammation and other symptomatic extensions during the dissemination phase [32]. It is generally accepted that the joints are mainly affected by RA. The basic pathological change in RA is synovitis. In the acute phase, the synovium shows exudative and cellular infiltration, with dilatation of small blood vessels in the sub-synovial layer, swelling of endothelial cells, and enlargement of the cellular spaces. While in the chronic phase, the synovium becomes hypertrophic and forms many villous protrusions that protrude into the joint cavity or invade into the cartilage and subchondral bone, which, if left untreated, can lead to joint destruction and disability [33]. The mechanistic links between RA and the inflammatory response are extremely complex, involving multiple dimensions of aberrant immune cell activation, cytokine network imbalance, and autoantibody-mediated tissue damage [34,35,36].

The core target cells of RA are SFs and SMs [37]. Synovial tissue macrophages are recognized as pro-inflammatory in RA, with the ability to release a large number of inflammatory cytokines, including IL-1, IL-6, and TNF-α [38]. A multitude of immune and non-immune cells are activated by inflammatory factors, and a variety of tissue-degrading enzymes secreted by these cells contribute to the chronic pro-inflammatory, tissue-destroying, and persistent pain response in RA [39]. TNF-α emerges as the principal inflammatory regulator, functioning through both autocrine stimulation and paracrine induction to orchestrate the production of crucial pro-inflammatory cytokines, including IL-1 and IL-6 [40]. Activation of TNF-α/NF-κB signaling causes the upregulation of NF-κB targets in foot and ankle RA patients, affecting the control genes that regulate cell growth, death, and inflammation [41]. The dysregulated release of IL-1β can be detrimental and is involved in the progression and pathophysiology of several chronic inflammatory diseases, especially RA [42]. IL-6, as one of the multi-effector cytokines, is a linchpin of the effector thymus [43]. Synovitis and joint destruction can be promoted by IL-6 [44]. A Mendelian randomized study suggested that long-term suppressive treatment of IL-1 and IL-6 might reduce the proportion of people who develop RA [45]. An advanced microenvironment-activating probe can target RA by conjugation with active and anti-IL-6R antibodies, manifesting NIR-II fluorescence signals at the lesion, enabling highly sensitive RA diagnosis and real-time treatment monitoring. The combination of ROS clearance and IL-6 signaling blockade leads to a potent treatment effect and synergistic immunomodulatory effects that significantly alleviate RA symptoms and prevent joint destruction [46]. Macrophage abundance and activation in the inflamed synovium/pannus have been shown to correlate with disease progression in RA. Therefore, targeting macrophage activation appears to be an effective therapeutic measure for reducing local and systemic inflammation and preventing irreversible joint damage [47].

T cells are the major effector cells of cellular immunity and are essential for synovial inflammation and joint destruction. Many cytokines are produced by T cells that play a role in the immune response. They directly or indirectly mediate inflammation and regulate other types of immune cells [48]. The Th1/Th2 immune response imbalance is critical in RA pathogenesis [49]. Th1 cytokines, for example, IL-2 and IFN-γ, promote the inflammatory microenvironment in RA patients and play a supportive role in the joints. In contrast, Th2 cytokines such as IL-4 and IL-10 have a beneficial effect against RA [50]. Th1 cytokines were demonstrated in all samples of RA synovium. Meanwhile, Th2 cytokines were detected in approximately 90% of samples [51]. Sophora decoction is expected to be an effective drug for the treatment of RA by decreasing the levels of Th1 cytokines and increasing Th2 cytokines in T cells to modulate the imbalance in RA rats, which is likely to be accomplished through the NF-KB pathway [52]. The animal drug dilong in traditional Chinese medicine may also have a therapeutic benefit in RA by blocking the NF-kb pathway [53]. A 12-week observation study was conducted on 40 patients with RA. In the etanercept combined with MTX treatment group, RA activity was improved by normalizing the distribution of Th17, Treg, and their related cytokines [54]. RNA sequencing was performed on the synovial tissues of several RA patients before and after tumor necrosis factor treatment. The baseline characteristic of RA patients is the activation of immune pathways, but this activation weakens after treatment. Moreover, several peripheral inflammatory proteins exhibit a decrease that is consistent with the corresponding changes in synovial gene expression [55].

For decades, researchers have commonly categorized the immune response into a Th1- and Th2-biased response. Yet, the discovery of new helper T cell subsets and the plasticity of helper T cells have changed the previous paradigm, suggesting that the immune process in this disease is the coordinated action of multiple T cells [56]. Tfh cells are important in the pathogenesis of various autoimmune diseases, not only in RA, due to their ability to promote B cell development, germinal center formation of lymphoid organs, and antibody production. Abnormal proliferation and other functional changes in Tfh cells can lead to pathological processes such as autoantibody production and tissue damage [57,58].

**Figure 2:**
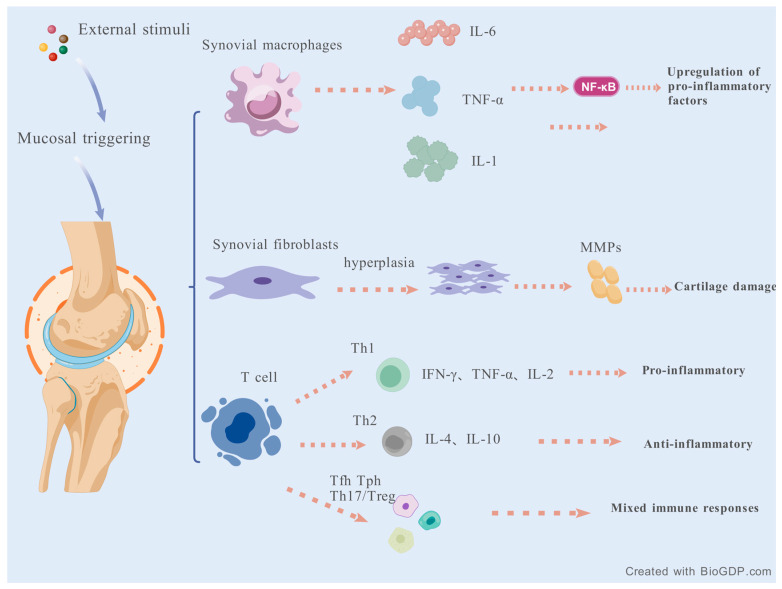
The role of immune cells and cytokines in RA.

Another cell, Tph cells, instead of expressing BCL6, expresses molecules like CXCL13, IL-21, and ICOS to support B cells in peripheral tissues [59,60]. The role of T-cells in the course of RA has become clearer as research into a variety of helper T-cells has deepened. Understanding the role of immune cells and cytokines in RA will help us provide more convenient and feasible strategies for the mechanism and efficacy of RA treatment in Figure 2.

### 2.2. Hypothalamic-Pituitary-Adrenal (HPA) Axis Disorders

The HPA axis has an essential regulating and controlling role in immune responses. Chronic stress promotes comorbidity of both the hypothalamus–pituitary–thyroid (HPT) axis and the immune system through dysregulation of the HPA axis [61,62]. The HPA axis plays a pivotal role in regulating and controlling the immune response. Disorders of this axis are among the causes of rheumatic diseases, including RA [63].

Bidirectional communication exists between the neuroendocrine and immune systems. These systems exert a reciprocal influence on one another, in accordance with physiological conditions and inflammatory stimuli [64]. In chronic inflammatory conditions, including RA, the HPA axis shows three changes: inappropriate cortisol release, low inflammation-related ACTH release, and decreased adrenal androgens [65]. Inflammation-associated inadequate cortisol production and decreased adrenal androgen production are features of HPA-associated endocrine findings in RA [66]. DHEA and DHEAS are the major adrenal AAs produced in humans. Differences in AA production occur when immune function is activated or abnormal, such as in RA and SLE, where DHEA and DHEAS are relatively low [67]. Research has indicated the presence of low levels of DHEAS in a specific group of women experiencing premenopausal RA [68]. The increased release of endogenous cortisol and growth hormone is a critical component of the metabolic response induced by TNF-α. In the RA patient, long-term treatment with anti-TNF therapy was found to sensitize the pituitary gland and improve adrenal androgen secretion [69]. Non-invasive vagus nerve stimulation (VNS) inhibits inflammation through the release of HPA and adrenocortical steroids and the CAP and increases vagal nerve activity [70]. Female patients appear to be more dependent on the HPA axis than men, and steroid production of adrenal androgens is more significantly altered in premenopausal RA patients treated with nonglucocorticoids [71]. Synovial macrophages, monocytes, and lymphocytes possess functional androgen and estrogen receptors, which may influence metabolism. They play a crucial immunomodulatory role in the context of sex hormone-related RA. Try to combine antirheumatic drugs such as MTX with androgen replacement therapy, and sex hormones can play a valuable concomitant or auxiliary therapeutic role [72]. The relationship between RA and the HPA axis is multi-layered, involving developmental programming, gene regulation, immune interaction, and stress feedback. A better understanding of this network could provide a novel strategy for treating psychiatric disorders, autoimmune diseases, and metabolic syndromes.

### 2.3. Autonomic Nervous System (ANS) Activation

Among numerous chronic autoimmune diseases, researchers have observed a common pathological state, namely the imbalance of the ANS. Naturally, this phenomenon has also been widely found in the population of RA patients [73]. Studies in recent years stand out that disruption of the ANS is associated with the onset and activity of RA [74]. The ANS deals with issues such as the supposed sympathetic/vagal balance, organization into plexuses, and “little brains” that are active, for example, in the enteric system or around the heart [75]. Targeting the nervous system can have the effect of reducing inflammation-related neurological symptoms while promoting immunity [76,77]. Forty-seven percent of RA patients and 19% of systemic lupus erythematosus (SLE) patients have symptoms indicative of autonomic nervous system dysfunction.

The characteristics of several mood disorders, including depression, are dysfunctions of the ANS (i.e., increases or decreases in sympathetic or parasympathetic activity). ANS activity is part of the central autonomic network involved in stress responses and is related to the pathophysiology of mood disorders [78]. In a retrospective cohort analysis, depression predicted RA progression. Patients with MDD (major depressive disorder) have a 38% increased risk of developing RA [79]. Antidepressants that have been demonstrated to be efficacious in the treatment of RA-MDD comorbidities, with the caveat that their administration should be accompanied by cognitive-behavioral therapy [80]. Immunoregulatory drugs (such as anti-TNF-α Ozoralizumab and anti-IL-6 tocilizumab RoActemra) improve anxiety and depressive symptoms in RA patients [80,81,82].

The vagus nerve, the primary parasympathetic nerve, in addition to its role in nutrient transportation, also has certain inflammatory and analgesic properties [83]. Transcutaneous auricular vagus nerve stimulation (taVNS) was observed to be effective in alleviating articular cartilage and bone destruction in CIA rats, which may be related to the reduction in osteoclast production, downregulation of MMP family expression levels, and RANKL/OPG ratio in rats with CIA [84]. Electrical or physiological (deep breathing-DB) VNS may be a potential treatment for disorders related to impairment of the ANS and vagus nerve function. For example, it is used to treat the common autoimmune diseases RA and systemic lupus erythematosus. DB (17–31%) and taVNS (18–25%), after all, the HRV parameters of heart rate variability (area) showed no difference between the two kinds of VNS [85].

Acupuncture is considered to be a reflexology that has an effect on the body’s ANS. Just as stated in the ancient book “Treatise on Cold Damage Disorders”, acupuncture using classical diagnostic procedures to assign acupuncture points to patients can alleviate pain in RA patients to some extent and improve hand function [86]. It is well known that exercise can partially restore the activity of the ANS, especially its parasympathetic nerve components. Patients with RA who are very inactive and/or at high risk of CVD may benefit substantially from a physical activity program [87]. A 12-week exercise program improved cardiac autonomic function in women with RA, as shown by short-term HRV, aligning with prior results [88]. Biofeedback of heart rate variability biofeedback (HRVB) is conducive to restoring the balance of the ANS and has a positive effect on emotional self-regulation. Mindfulness meditation on the mind could reduce autonomic nervous system reactivity [89]. A growing body of evidence highlights the effectiveness of non-drug approaches, including yoga, mindfulness practices, and emotion-focused therapies, in significantly alleviating mood disturbances and reducing inflammation in rheumatoid arthritis patients [90].

Recent breakthroughs in the study of immune-autonomic neural pathways, which reveal the intricate interplay between the nervous and immune systems, are opening doors to groundbreaking approaches for managing rheumatoid arthritis.

### 2.4. Anemia

Anemia is one of the common symptoms in RA patients [91]. Anemia is a prevalent hematological disorder characterized by decreased RBCs in the body’s peripheral blood [92]. This will lead to insufficient oxygen supply to tissues and organs [93,94]. A multi-organizational cross-sectional observational study in tertiary care hospitals showed that in the study of 330 patients with RA seen from January 2023 to December 2023, 180 patients (54.55%) developed anemia [95]. A potent clinical association between anemia and RA has been confirmed by logistic regression analysis. Using GWAS analysis, the overall genetic correlation of the two diseases was positive, which confirmed that rheumatoid arthritis has a significant causal relationship with anemia [96]. To sum up, it is not difficult to find that anemia is a frequent symptom in RA patients. Anemia has also been shown to have deleterious effects on the cardiovascular system, the CNS, and the kidneys [97].

There are many causes of anemia, and the popular belief is that it is related to nutritional deficiencies (e.g., iron), blood loss, and hereditary disorders (e.g., thalassemia) [98]. Unlike general anemia, RA anemia is associated with inflammatory factors [99]. The pathological causes of combined anemia in patients with RA are mainly related to chronic inflammation and immune abnormalities, with the central mechanism being anemia of chronic disease (ACD) [100]. The chronic inflammatory state in RA patients leads to a massive release of pro-inflammatory cytokines, including IL-6, which interfere with iron metabolism and erythropoiesis, leading to anemia of inflammation (AI) or ACD [101,102]. Inflammatory factors inhibit renal erythropoietin (EPO) synthesis and decrease bone marrow responsiveness to EPO. IL-6 upregulates hepatic secretion of ferroportin (hepcidin), which inhibits intestinal iron absorption and macrophage iron release, resulting in impaired iron metabolism [103]. Meanwhile, oxidative stress and inflammation accelerate erythrocyte destruction. Inflammatory factors (e.g., TNF-α) directly inhibit the proliferation of red lineage progenitor cells, leading to decreased erythropoiesis [104].

In addition, there are many causes of RA anemia, such as disease medications and inflammatory diets. Many drugs are now often used clinically to treat RA, such as methotrexate, which may directly suppress bone marrow hemopoiesis, leading to megaloblastic anemia or pancytopenia [105]. Immunosuppressive agents such as azathioprine may cause bone marrow suppression, leading to pancytopenia [106]. Long-term use of NSAIDs may also trigger occult bleeding in the gastrointestinal tract and aggravate iron deficiency anemia [107]. In a 6-month-long study of celecoxib versus omeprazole and diclofenac in the Treatment of Patients with High-Risk Osteoarthritis and RA Pilot Study and the Long-Term Arthritis Safety Study of Celecoxib, hemoglobin was reduced by ≥2 g/dL in 1.9% and 2.0% of celecoxib-treated and 3.3% and 5.7% of diclofenac-treated patients in the two trials, respectively [108]. According to the latest WHO criteria, a columnar graphical model of key dietary factors showed that a total of 10.25% with RA developed anemia, and furthermore, anemic patients had higher levels of dietary inflammation index (DII) than non-anemic patients [109].

### 2.5. Thyroid Abnormalities

The connection between RA and the thyroid gland has long been extensively studied, focusing on a) functional and immune thyroid abnormalities with a prior history of RA and b) joint changes with prior antithyroid drug (ATD) [110]. Research has revealed a reciprocal causal link between thyroid function and RA. Genetic susceptibility to hypothyroidism appears linked to a higher risk of RA. Similarly, a link has been suggested between RA and hypothyroidism, as well as other types of hyperthyroidism [111]. The interaction between the thyroid gland and RA is a combined result of an imbalance in the immune–endocrine–metabolic network. A vicious circle is formed by genetic background, inflammatory factors, hormone axis interactions, and environmental factors [112,113,114].

NHANES data (2009–2012) suggest inflammation influences thyroid dysfunction onset and progression. Abnormal thyroid hormone levels can affect immune cell function promptly. Early anti-inflammatory intervention may mitigate thyroid irregularities [115]. The elevated T3 level in patients with hyperthyroidism promotes Th1 cell differentiation and tends to exacerbate the autoimmune response of RA. Similarly, in patients with hypothyroidism, insufficient thyroid hormone weakens regulatory T cell (Treg) function, leading to insufficient immunosuppression, which may exacerbate RA [111,116]. In the early stage, there is an obvious cross-talk between antigen-presenting cells and Th cells. As the disease progresses to the advanced stage, antigen-specific and non-specific immune cells migrate to the thyroid, forming inflammatory infiltrates [114]. The incidence of thyroid dysfunction is at least three times higher in women with RA than in women with non-inflammatory rheumatic diseases like osteoarthritis and fibromyalgia [117].

RA and autoimmune thyroid disorders could share a genetic connection. Of the 58 available screened members of 504 RA multi-case families, 6% of patients had thyroid disease, thyroglobulin antibodies were present in 5% of men and 11% of women, and thyroid microsomal antibodies were present in 5% of men and 15% of women. Clearly, these rates are higher than the general level of published data. More strikingly, this difference persisted after rigorous analysis of the age groups individually [118].

Medications and treatments may also affect the course of the disease. For example, methotrexate is effective in RA therapy and may improve the thyroid autoimmune response. However, long-term use will affect thyroid hormone metabolism to a certain extent, and the patient’s liver function needs to be monitored [119]. In addition, diet and lifestyle habits also play a role. Daily vitamin D intake is associated with elevated RA disease activity and a higher prevalence of thyroid autoantibodies [120]. Anti-TNF-α and anti-IL-17 therapies may be considered as reasonable treatment options for thyroid-related disorders [121].

### 2.6. Other Mechanisms

Enrichment analysis of the intersecting targets of anemia and RA targets resulted in highlighting pathways associated with inflammation, immune response, and iron metabolism. Further testing by SMR analysis showed that BLK and FAM167A were ranked as the top two most promising genes [96]. The regulation of iron homeostasis in RA is impaired, and dysregulation of iron balance can contribute to disease progression via mechanisms including the generation of ROS, fibrosis, inflammation, abnormalities in bone homeostasis, and cellular senescence [122]. Iron-induced anti-inflammatory macrophage M2 ferroptosis is highly susceptible, while pro-inflammatory M1 is to a lesser extent affected. Different GPX4 degradation in both cell types depends on p62/SQSTM1 and contributes greatly to progression [123].

In a pro-inflammatory environment, T cells and macrophages switch from oxidative phosphorylation to glycolysis (Warburg effect), resulting in a decrease in ATP production efficiency. When ATP is decomposed into ADP/AMP, the release of free energy decreases, which affects energy-consuming processes such as ion pumps and protein modification, resulting in cell dysfunction [123]. The reduction of adenosine, the ATP breakdown product, weakens its anti-inflammatory effects mediated through A2A receptors [124].

In addition to the above-mentioned pathogenesis, the pathogenesis of RA involves many layers and multi-level interactions and does not exist in isolation. Further work is required in the future to clarify the specific molecular functions of these pathways and explore their potential for clinical translation.

## 3. Current Research Progress of the RA Model

For better exploration of the pathogenesis of RA and the pharmacological effects of traditional Chinese medicine on RA, it is important to choose a suitable RA model construction method [125]. Animal models facilitate the study of RA, thereby enabling researchers to study complex systems involving inflammation, immune tolerance, and autoimmunity [126]. In addition to some conventional surgical modeling methods, the latest experimenters used some emerging methods.

Studies show that sinomenine reduces collagen-induced arthritis by stopping new blood vessel formation in mice. This supports its use in RA treatment [127]. An attempt was made to investigate the relationship between dietary patterns, circadian oscillations of the gut microbiota, and RA rhythmicity in CIA mice and RA patients using dietary timing-induced diurnal oscillations of the gut microbiota.

This showed that Parabacteroides distasonis influences RA inflammatory rhythms based upon the SIRT5-NF-κB axis [128]. CAIA modeling is emerging at the beginning of the 21st century and is particularly suitable for rapid pharmacodynamic evaluation [129]. In comparison with the original CIA model, the CAIA model has been demonstrated to elicit arthritis in the majority of mice, including those that are CIA insensitive. Moreover, CAIA has a short modeling period, accelerating the screening and evaluation of therapeutic agents for RA [130]. The model can be extended to transgenic mice to resolve the mechanisms of gene regulation of arthritis progression, as well as to support the systematic assessment of the function of RA-associated inflammatory mediators and causative factors such as microbial toxins [131,132]. Human SCID transplantation is a common technique for the long-term study of RA explants [133]. This model allows for the measurement of long-distance migration by synovial cells in human RA and these cell-induced cartilage destruction allows for the initial screening of human target-specific biologics [134].

In addition to the above chemical and physical methods for model construction, transgenic mice with self-deficiencies are also widely used in RA research. CIA modeling of transgenic mice lacking CD4 and CD8 molecules expressing the RA susceptibility gene HLA-DQ8 shows that CD4-deficient mice are resistant to CIA, CD8-deficient DQ8 transgenic mice have an increased susceptibility to CIA and auto-antibody development, and CD8(+) T cells fail to initiate CIA in the DQ8 transgenic mice but may have a regulatory/protective role [135]. Susceptible mice are an invaluable tool in mimicking the multifactorial pathogenesis of RA, inducing arthritis through microbial stimulation, which is closer to the complex etiology of human RA [136]. Abhinav Lamba et al. used mice expressing RA susceptibility and drug-resistant HLA class II genes to describe their interactions with gut dysbiosis in preclinical autoreactivity of sex bias [137]. The construction of more authoritative and efficient RA models will provide more convenience and feasible strategies for the investigation of the mechanism and efficacy of TCM in RA treatment.

## 4. The Potential Pharmacological Mechanism of SAL in Anti-RA

### 4.1. Improve Exercise Endurance

*Rhodiola rosea* improves endurance and fatigue during exercise. Acute and sustained 4-week supplementation with SAL in competitive rowers increased plasma antioxidant levels but did not affect exercise-induced oxidative damage [138]. Another double-blind experiment, which was a study investigating the impact of an acute intake of *Rhodiola rosea* on physical performance, muscle strength, and limb movement speed in athletes, was found to achieve an increase in endurance exercise capacity in volunteers [139]. In a detailed experiment investigating swimming-induced oxidative stress in rats, treatment with *Rhodiola rosea* (5, 25, 125 mg/day/rat) extract for 4 weeks significantly suppressed the production of O_2_(-)* in the blood and liver. Additionally, it increased the concentration of malondialdehyde in plasma. The four primary components of the extract, including SAL, were found to effectively eliminate O_2_(-)*, decrease the activity of H_2_O_2_ and HOCl, and exhibit a certain degree of dose dependence. The experiments further revealed that the expressions of Mn-superoxide dismutase, Cu/Zn-superoxide dismutase, and catalase were all upregulated in the livers of the rats. These findings collectively suggest that oxidative stress may be alleviated through the scavenging of reactive oxygen species and enhancement of antioxidative capacity by SAL [140].

### 4.2. Energy Metabolism Effects

#### 4.2.1. Glycogen Storage Effects

SAL may exert its anti-fatigue effect by regulating cellular energy metabolism. In the weight-bearing experiment conducted on mice, SAL treatment (100 mg/day/rat) was found to markedly prolong swimming endurance compared to the control group (26.2 min vs. 10.5 min). Furthermore, the activity of mitochondrial respiratory chain enzyme complexes in the skeletal muscle tissue of SALt-treated mice was significantly increased [141]. SAL (0.1, 1, 10 μM) significantly downregulated LOX-1 and upregulated ATP-binding cassette transporter A. Furthermore, SAL significantly decreased the phosphorylation of JNK, ERK, and p38 MAPK, while increasing the phosphorylation of Akt, which resulted in an inhibition of oxidative LDL-induced formation and apoptosis of THP1-derived foam cells [142].

#### 4.2.2. Promote Lipid Metabolism

SAL is not simply about “lowering blood lipids,” but rather involves a dual regulation of “immune and metabolism”. By reprogramming lipid metabolism, redox metabolism, and energy metabolism in both immune cells and metabolic organs through multi-system and multi-target approaches, SAL can correct pathological and pro-inflammatory metabolic conditions, restoring a healthy and stable metabolic state. SAL reduces lipid accumulation in macrophages and atherosclerotic plaques by directly binding to Hif-1α and inhibiting the thermal protein deposition pathway. SAL significantly reduces lipid accumulation in macrophages in a dose-dependent manner (EC50 = 50.49 μM) [143]. SAL demonstrates significant therapeutic effects by suppressing CD8 T cell infiltration, mitigating oxidative stress, and alleviating inflammatory factors. This multifaceted approach enhances mitochondrial metabolism, iron metabolism, lipid metabolism, and redox metabolism in the brains of SAMP8 mice [144]. Low-dose SAL (0.3 mg/mL) can inhibit the generation of ROS and lipid accumulation in the fatty liver model of laying hens in vitro through the PI3K/AKT/Gsk3-β pathway. Higher doses of SAL (20 mg/kg) can alleviate liver steatosis in laying hens induced by a high-fat diet [145]. SAL alleviates lipid accumulation and inflammatory response in primary hepatocytes after palmitic acid/oleic (OP) acid stimulation and can inhibit the progression of NASH induced by metabolic stress by activating AMPK signal transduction [146]. One hundred micromoles of SAL greatly increased the subsequent blastocyst formation rate and embryo quality of porcine oocytes, oocyte lipid content, and mitochondrial number [147]. SAL (30 mg/kg/day, 60 mg/kg/day) reduces the levels of ROS and LPO in the oxidative stress model induced by t-BHP. Further research and analysis in network biology and cell metabolomics indicate that galactose metabolism and fatty acid metabolism are the major ways in which it functions [148].

#### 4.2.3. Repair Mitochondrial Dysfunction

Scientists have conducted various experiments to explore the potential mechanism of *Rhodiola rosea* in treating metabolic disorders. The mechanisms involved include multi-target effects that regulate oxidative stress, inflammation, mitochondria, autophagy, cell death, etc. They work together through multi-pathway and multi-level synergistic pathways [149]. The mitochondrial dysfunction is closely related to RA [150]. Imbalance of mitochondrial homeostasis is closely associated with cardiovascular disease, and SAL was found to have beneficial anti-inflammatory and anti-atherosclerotic effects, suggesting that SAL has potential in mitochondrial treatment for RA [150]. SAL intervention reduced IL-1β and TNF-α mRNA expression and inhibited the changes in mitochondrial membrane potential and mass, ROS level and nuclear translocation of NF-κB p65, but had no effect on the expression of iNOS. This finding demonstrates that SAL (400 μM) effectively counteracts M1 macrophage polarization; it accomplishes this by enhancing mitochondrial membrane potential while modulating the expression of both pro-inflammatory mediators and proteins critical to mitochondrial homeostasis [151]. SAL (100–400 μg/mL) effectively curbed melanoma growth by disrupting key cellular processes, including proliferation and migration. It also impaired metabolic functions by reducing both glycolysis and oxidative phosphorylation [152].

### 4.3. Regulate Amino Acid Metabolism

The regulation of amino acid metabolism by SAL is one of the important mechanisms by which it exerts its anti-RA effect. SAL demonstrates its potential as an effective anti-RA drug by regulating amino acid metabolism, alleviating endoplasmic reticulum stress, and correcting metabolic disorders caused by RA. Studies have shown that there are significant amino acid metabolic disorders in RA model mice [153]. Metabolomic profiling of CIA mouse plasma revealed a significant reduction in 20 out of 45 detected amino acid metabolites, linking these metabolic perturbations to RA-induced muscle atrophy and energy imbalance [154]. Pretreatment with SAL (1, 3, 30, 300 μmol/L) demonstrated a dose-dependent protective effect against Hcy-induced cytotoxicity in HUVECs. It primarily works through suppressing the expression of ER stress markers, including Bip and CHOP, as well as inhibiting the phosphorylation of PERK and IRE1α [155]. *Rhodiola rosea* extract (200 μg/mL) has been identified as a novel tyrosinase inhibitor; the inhibitory mechanism of it is mediated through modulation of the CREB/MITF/tyrosinase signaling pathway in B16F0 melanoma cells [156]. This also reflects from another perspective the potential of SAL and its source extracts in regulating amino acid metabolism-related signaling pathways.

However, the limited availability of plant sources restricts its large-scale production. To address this challenge, microbial biosynthesis of SAL via heterologous expression of plant-derived uridine UGT enzymes presents a viable and sustainable alternative [157].

### 4.4. Reduce Metabolite Accumulation

SAL has significant anti-fatigue properties, mechanistically linked to attenuating oxidative stress damage, reducing harmful metabolite accumulation, and increasing energy substrate stores. The metabolic signature of RA extends beyond energy provision to actively participate in disease pathogenesis by: (i) fueling hyperplastic synovial growth, (ii) maintaining pro-inflammatory cytokine production, (iii) recruiting immune cells to joints, (iv) activating osteoclast-mediated bone erosion, and (v) disrupting protein homeostasis in skeletal muscle, collectively contributing to RA etiology [158]. Samples were taken from the muscles of RA patients, and mRNA targeting measurements of the samples confirmed that both muscle energy metabolism and metabolic gene expression are modified within a CIA model, and RA contributes to dysregulation of metabolic homeostasis in skeletal muscle [159]. An important signaling pathway that could link inflammatory processes and RA is IDO. IDO is induced by inflammatory stimuli and catalyzes the catabolism of tryptophan to kynurenine for subsequent conversion to neuroactive metabolites [160]. In comparison with the model control group, both the *Rhodiola rosea* capsules and SAL treatment groups (5–20 mg/kg) exhibited a statistically significant prolongation of exhaustive swimming time in mice. Biochemical analyses revealed that SAL treatment effectively mitigated oxidative stress, as evidenced by decreased levels of MDA, H_2_O_2_, and lactic acid in hepatic and muscular tissues, concurrent with increased concentrations of GSH, hepatic glycogen, and muscle glycogen. Furthermore, SAL administration significantly enhanced the activities of total superoxide dismutase (T-SOD) and ATPase [161].

### 4.5. Antioxidant Activity

Oxidative stress occurs in many autoimmune diseases, including RA, along with overproduction of ROS and RNS; thus, antioxidant treatment appears to be the most valuable therapeutic approach [162]. SAL has significant antioxidant activity [163]. In vitro antioxidant studies also proved that it has excellent antioxidant properties [164]. By means of computer-aided methods and label-free interaction analysis, it was found that SAL directly inhibits HSP90, inhibits its ATPase activity, and affects the interaction of related proteins and the expression of downstream genes, effectively prolonging lifespan and regulating oxidative stress, and exerting anti-aging and antioxidant properties [165]. SAL (20, 40 mg/kg) protects against LPS-induced cardiac injury by targeting iNOS/COX-2 and NF-κB/PI3K/Akt/mTOR pathways. NAC pretreatment in H9C2 cells verified ROS-dependent mechanisms, supporting SAL’s potential for cardiovascular therapy [166].

People with RA may develop some brain disorders. Inflammatory factors produced during the RA pathological process, like IL-17, may affect the stroke process [167]. Endothelial cell function may be impaired, and endothelial dysfunction and cranial nerve barrier (BNB) may be pathological for RA patients [168]. In MCAO-induced CIRI rats after SAL treatment (50, 100 mg/kg), there was a significantly reduced infarction rate and improved histopathological changes. SAL was also effective in inhibiting apoptosis and reducing the level of oxidative stress in PC12 caused by OGD/R stimulation. The alleviation of apoptosis may be due to enhanced inhibition of cleaved caspase-3 and Bax/Bcl-2 proteins and also reduced MDA formation, and oxidative stress was strongly associated with upregulation of SOD and CAT activities and Nrf2 and Trx1 expression [169]. SAL (0.1,1,10 nM) possesses antioxidant functions and attenuates H_2_O_2_-induced apoptosis in cells, which is closely related to the activities of IGF1R and *p*-ERK1/2 [170].

SAL (0, 200, 400, and 600 mg/kg) enhances the activity of antioxidant enzymes and upregulates the expression of antioxidant genes in both the liver and muscle [171]. SAL (20 mg/kg) reduces reactive oxygen species levels, decreases malondialdehyde levels, and enhances superoxide dismutase and catalase protein expression in the ipsilateral testes, thereby ameliorating testicular ischemia-reperfusion injury by upregulating superoxide dismutase and catalase protein expression [172]. SAL (5,10,20μM) increases antioxidant capacity by lowering MDA, raising SOD, CAT, and GSH, and scavenging mitochondrial ROS. The salvage of mitochondrial ultrastructure, retention of MMP and ATP, inhibition of cytoplasmic cytochrome and cleaved caspase 3 expression, and inhibition of apoptosis suggest that *Rhodiola rosea* glycoside is a mitochondria-targeted antioxidant [173]. SAL has achieved remarkable results in neurological diseases because of its antioxidant activity.

**Figure 3:**
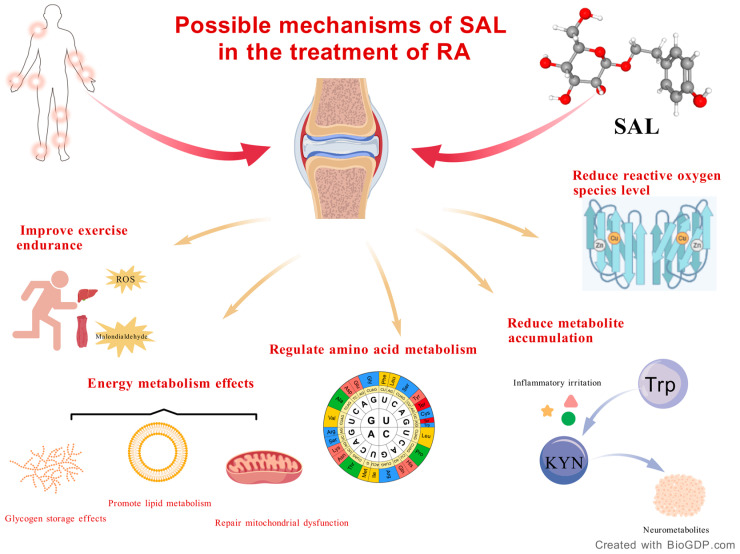
Possible mechanisms of SAL in the treatment of RA.

SAL may have anti-RA effects, which are mediated by multiple pathways like antioxidant stress and reduced metabolite accumulation, as shown in Figure 3.

### 4.6. Regulate Immune System

Immune system imbalances are thought to be connected with the symptoms of RA [174,175,176]. Network pharmacology and experiments have shown that GSZD (Guizhi-Shaoyao-Zhimu) may be able to partially reverse the imbalance of the inflammatory-immunomodulatory network that occurs during the progression of RA by modulating the HDAC1-HSP90AA1-NFKB2-IKBKB-TNF-α axis [177]. The CIA rat model and the TNF-α-induced HFLS-RA model were established. The Rosea-euonymus alatus drug of *Rhodiola rosea* can inhibit the PI3K/AKT signaling pathway and regulate the balance between matrix metalloproteinases and their inhibitors, thereby exerting therapeutic effects in RA [178]. After further integration with LC-MS/MS and bioinformatics, it was found that the onset of RA may be related to inflammation-associated immune cells involved in adaptive and innate immune responses. The potential key targets for treatment include seven, such as AKR1B10, MMP13, and FABP4. Further component research identified salidroside as one of the key components [179]. Comprehensive network pharmacology and experimental methods were explored to investigate the therapeutic mechanism of Jingulian capsules on RA, and JGL included the active ingredient of SAL that can inhibit inflammation through the IL-17/NF-κB pathway, thereby showing a protective effect on RA [180].

Pharmacological studies revealed that the inhibitory effect of SAL on BMDM pyroptosis was partially attenuated by TREM1 inhibition. In the context of adaptive immunity, SAL treatment restored gut microbiota diversity in DSS-induced colitis mice while rebalancing the Th17/Treg cell ratio. SAL ameliorates experimental colitis through dual modulation of macrophage proptosis and Th17/Treg homeostasis, highlighting its therapeutic potential for related immune disorders, like RA [181]. In addition to joint damage, RA can also have extra-articular manifestations, such as liver damage [182]. SAL demonstrates significant hepatoprotective properties. In bleomycin (BLM)-induced pulmonary injury models, characteristic EMT markers were observed, including decreased E-cadherin expression and upregulation of mesenchymal markers. Mechanistically, SAL (20, 40 mg/kg) treatment effectively attenuated these BLM-induced EMT alterations by enhancing the expression of TGF-β1 and phosphorylation of its downstream signaling molecules Smad-2/3 [183,184]. SAL (20 mg/kg/day) also improves hepatocyte death and apoptosis by activating GSK-3β/NrF2-dependent antioxidant responses and subsequently inhibiting MPTP [185].

Anemia is a significant symptom of RA [186]. The pathogenesis of acquired aplastic anemia (AA) is tightly correlated with the imbalance of Th17/Treg cells, and severe AA can even lead to death. The elevated level of HIF-1α in patients with AA constitutes a relatively important pathological feature. In the SAL group, blood cell counts were increased, bone marrow function was improved, the number of blood cells showed an increasing trend, the expression of FoxP3 was increased, and the imbalance of Th17/Treg was corrected. Meanwhile, in the SAL group, STAT3, RORγt, and IL-17a showed a downward trend, and the upward trend of HIF-1α level was also reversed. SAL corrects the Th17/Treg imbalance through the STAT3/HIF-1α/RORγt pathway and has the potential to be a novel therapeutic drug for AA and RA [187]. SAL can serve as an adjuvant for red blood cell production, effectively addressing anemia and hypoxia. SAL has been shown to enhance erythropoiesis in EPO-treated cells and decrease the rate of apoptosis in TF-1 erythroblasts following H_2_O_2_ treatment. This protective effect may be mediated through the upregulation of antioxidant defense proteins, specifically thioredoxin-1 (Trx1) and glutathione peroxidase-1 (GPx1) [187]. The SAL (75 μg/g body weight) activation of PARP-1 can affect the homeostasis and function of hematopoietic stem cells in mice under oxidative stress and prevent the loss of hematopoietic stem cells (HSCs). However, SAL does not prevent the production of reactive oxygen species but reduces the DNA strand breaks induced by hydrogen peroxide in hematopoietic stem cells [188]. SAL may also promote the recovery of hematopoietic function in mice by terminating G0/G1 phase arrest, accelerating the transitions between G0/G1-S and S-G2/M phases, upregulating Bcl-2 expression, and downregulating Bax expression [189].

**Figure 4:**
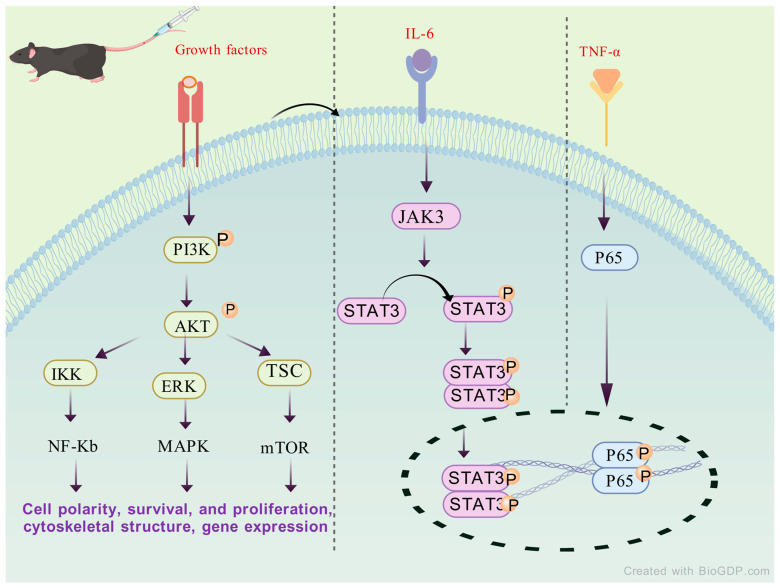
The potential mechanism of SAL acting against RA by regulating STAT3 signaling.

The inhibitory effect of SAL on the IL-17/NF-κB and STAT3 pathways suggests that SAL may have therapeutic value in related diseases, based on the multiple central and peripheral effects of inflammatory factors in Figure 4.

### 4.7. Interfere with the Synthesis and Release of Neurotransmitters in the Brain

SAL demonstrates broad neuroprotective potential in various neurological disease models by synergistically exerting anti-inflammatory, antioxidant, and neuroregulatory effects. The inflammatory response induced by LPS was significantly reduced after pretreatment with SAL (12 mg/kg and 24 mg/kg). The expression levels of BDNF and TrkB increased, with a decrease in NE and 5-HT levels in the prefrontal cortex, suggesting that SAL exerts neuroprotective effects via the BDNF/TrkB signaling pathway [190]. The pathogenesis of attention deficit disorder is hypothesized to be associated with Aβ-induced oxidative stress and chronic neuroinflammation in the brain. This point is similar to the pathogenesis of RA in terms of inflammation and oxidative stress [191]. Oxidative stress mediated by NADPH oxidase in rats induced by Aβ_1-40_ increased, NF-κB was in an activated state, and the expressions of related inflammatory factors such as COX-2 and iNOS were significantly upregulated. SAL (25, 50, and 75 mg/kg *p*.o.) treatment group reversed all the aforementioned changes [192]. SAL (50 mg/kg) treatment activates the Nrf2-ARE signaling, inhibits oxidative stress and neuroinflammation, and achieves certain therapeutic effects in rats [193]. SAL ameliorates isoflurane-induced cognitive dysfunction by inhibiting TNF-α and IL-1β release, reducing oxidative stress, and restoring cholinergic balance in the hippocampus, such as acetylcholine and superoxide dismutase [194]. SAL) (60, 120, 180 mg/kg) has stress-protective properties that could also improve cognitive functions by inhibiting caspase-3 activity and antagonizing NO and NOS production during H_2_O_2_ stimulation [195].

After pretreatment of PC12 cells with SAL, the data showed that apoptosis was significantly reduced and the collapse of mitochondrial membrane potential was alleviated. Meanwhile, SAL (10,50,100μM) inhibits the increase in NO induced by MPP (+) and the overexpression of nNOS and iNOS and suppresses the accumulation of ROS and intracellular free Ca^2+^, disrupting key mediators of neurotoxic signaling cascades [196].

The iminodipropyl nitrile (IDPN)-induced Tourette syndrome model (TS) was created. Compared with the positive drug haloperidol treatment group, the tic behavior score in the SAL (0.5 mg/kg × d^−1^) treatment group increased, the DA level in plasma and striatum decreased, and the 5-HT level increased, resulting in a marked rebalancing of central neurotransmitter levels. Therefore, the therapeutic mechanism of SAL (50 mg/kg/d) is likely to be associated with the modulation of DA neurons in the striatum, potentially through presynaptic regulation or receptor interactions [197]. Long-term chronic stress can activate the peripheral and central immune systems, triggering the release of a large number of inflammatory cytokines, which is one of the important pathophysiological mechanisms for the occurrence of depression [198,199].

Both depressive states and inflammatory responses can lead to excessive generation of reactive oxygen species, thereby triggering oxidative stress and further damaging lipids, proteins, and DNA in neurons [200,201]. SAL (30, 90 mg/kg and 0.1 μg/mL) itself is an effective antioxidant that can activate the core antioxidant defense pathway Nrf2/ARE [202,203] in the body and may increase the availability of 5-HT, NE, and DA in the synaptic cleft through other indirect mechanisms (such as reducing inflammatory responses to improve neurotransmitter function), thereby improving emotional state, enhancing motivation, and restoring pleasure experience [190,204]. In traditional Chinese medicine, *Rhodiola rosea* is utilized to invigorate qi and promote blood circulation, demonstrating potential therapeutic effects in the treatment of depression. SAL (at least 20 mg/kg) stimulates hippocampal neurogenesis through the upregulation of the SIRT1/PGC-1α signaling pathway, inhibits the P2X7/NF-κB/NLRP3 axis to regulate NLRP3 inflammasome-mediated proptosis, reduces the expression of pro-inflammatory cytokines such as IL-6 and TNF-α, suppresses microglial activation, enhances monoaminergic neural transmission, and attenuates LPS-induced neuroinflammation [205].

SAL may have a therapeutic effect on RA through the above-mentioned mechanism. The detailed mechanism and the involved models are shown in Table 3.

## 5. Pharmacokinetics, Safety and Toxicity

After intravenous administration of SAL (7.5–30 mg/kg) in rats, it exhibited linear pharmacokinetics with a short t1/2 < 1 h and no sex-related differences. SAL was widely distributed within 15 min, with the highest levels in the liver, adipose tissue, skeletal muscle, ovary, and testis. Renal clearance was the major elimination pathway, with 53.67% of the dose excreted unchanged in urine within 48 h. In contrast, fecal and biliary excretion were minimal (<2%). The low total recovery (<54%) indicates significant metabolic clearance [206]. Salidroside underwent more extensive metabolism following gavage administration compared to intravenous (i.v.) administration. The total excretion rates of salidroside, including both salidroside itself and its metabolite *p*-tyrosol, were 68.85% after i.v. administration and 26.07% after gavage administration [207]. Rats were orally administered 12–48 mg/kg SAL for plasma concentration determination. The highest intestinal absorption was achieved at an oral dose of 24 mg/kg [208]. The method was utilized in a pharmacokinetic study of salidroside injection conducted on six beagle dogs. Following a single intravenous dose of 75 mg/kg, the primary pharmacokinetic parameters of salidroside in the dogs were as follows: Cmax was 96.16 ± 8.59 µg/mL, AUC0–24 was 180.3 ± 30.6 µg·h/mL, AUC0–∞ was 189.3 ± 32.1 µg·h/mL, and the half-life (t1/2) was 2.006 ± 0.615 h [209].

*Rhodiola rosea* polysaccharides, as a kind of natural polymer material, are characterized by their non-toxicity and non-irritation. They are often used as key components in drug formulations and made into various dosage forms such as injections and capsules [210]. In the safety evaluation of SAL in the treatment of viral myocarditis, including (bacterial reverse mutation test, chromosomal aberration test, and mouse micronucleus determination), no structural and numerical changes or increase in chromosomal aberration were observed at all treatment doses (500, 1000, and 2000 μg/kg) [211]. Administration of a 20 mg/kg body weight aqueous-alcoholic extract of *Rhodiola rosea* to pregnant and lactating mice may modulate prenatal and postnatal angiogenesis, potentially involving additional unidentified bioactive compounds that influence endothelial cell proliferation [212].

Based on the above mechanism, we speculate that the combination of salidroside and the following drugs may have adverse effects. *Rhodiola rosea* is known in traditional Chinese medicine for its properties of promoting blood circulation and resolving blood stasis [213]. Its concurrent use with anticoagulant medications, such as warfarin, may increase the risk of abnormal bleeding. Additionally, due to the presence of salidroside, which may influence the levels of monoamine neurotransmitters such as HT and NE [190], it is not recommended to use *Rhodiola rosea* in combination with monoamine oxidase inhibitors (MAOIs), including older antidepressants like phenylhydrazone and trans-phenyl cyclopropylamine. *Rhodiola rosea* is widely recognized for its adaptogenic properties and is commonly used to alleviate fatigue [190]. However, when taken alongside sedative-hypnotic drugs such as diazepam and barbiturates, it may exert antagonistic effects.

## 6. Conclusion and Perspectives

*Rhodiola rosea*, as a traditional Chinese medicinal herb, has a sweet, bitter, and neutral taste. It enters the lung and heart meridians. Thousands of years of clinical experience in traditional Chinese medicine have concluded that it has the effects of benefiting qi, promoting blood circulation, unblocking meridians, and relieving asthma. It is used in TCM to treat qi deficiency, blood stasis, chest pain, and heart pain. *Rhodiola rosea* has long been listed as a superior medicinal herb in the “Shennong’s Classic of Materia Medica” and is widely used in China and other Asian countries, benefiting the world. Phenolic compounds such as rhodioloside and tyrosol glucoside are responsible for *R. rosea*’s biological effects: antioxidant, immunomodulatory, antiaging, and anti-fatigue [214]. SAL has powerful antioxidative, anti-inflammatory, cancer-fighting, and age-fighting properties and has been used in the cosmetics industry [215,216]. In the medical field, the huge potential of SAL in multiple aspects, such as antioxidation, makes its development into immunomodulatory and anti-RA practical and promising.

In addition to its significant antioxidant effects, SAL also has strong anti-inflammatory effects. The strong anti-inflammatory effect has also become a significant advantage of SAL in treating RA symptoms.

Therefore, in the future, we can design a novel RA-targeted nano-preparation to directly exert a therapeutic effect at the site of RA lesions and, at the same time, achieve the purpose of alleviating inflammation, so as to achieve the dual therapeutic effect [217].

To summarize: SAL holds significant potential for application in the clinical treatment of RA. Nevertheless, further exploration and validation of its anti-RA mechanism are required through large-scale, systematic, and standardized preclinical animal experiments, as well as extensive clinical trials.

## Figures and Tables

**Table 1:** SAL versus the current RA medication.

	smDMARDs	tsDMARDs	PIs	SAL
Chemical nature	Artificially synthesized small molecules	Biological macromolecules (antibodies)	Artificially synthesized peptide small molecules	Small molecules of natural plant origin
Mode of operation	Inhibit a single kinase or enzyme	Neutralize single cytokines	Inhibit the intracellular proteasome complex	Regulate the core signal hub (NF-κB, Nrf2)
Advantage	Low cost, exact efficacy, basic drug	High potency, strong specificity, oral convenience, quick action	Can induce proinflammatory cell apoptosis	Multiple benefits (anti-inflammatory + antioxidant), high safety potential, natural source
Disadvantage	Non-specific and with many side effects	Immunogenicity, high cost, and low selectivity	High toxicity (neuropathy, cytopenia	Slow onset, high dose, and complex mechanism

**Table 2:** Possible mechanisms involved in RA.

Whole Body System	Specific Mechanism
Endocrine system	Thyroid abnormalities [19]
Central nervous system	HPA axis function, neuroinflammation, and the gut microbiota–gut–brain axis [20]
Peripheral nervous system	Stimulation of the vagus nerve via inflammation [21]
Metabolic function	Decreased aerobic exercise [22] Metabolite accumulation [23] Decreased free energy of ATP breakdown [24] Mitochondrial dysfunction disorders [25]
Immune system	Changes in inflammatory factor levels [26] Th1/Th2 cell imbalance [27]
Depression	Overexpression of the main iron regulatory hormone hepcidin [28]
Oxidative stress	Oxidative stress [29]
Others	Viral infection; circadian rhythm disorder [30]

**Table 3:** Possible mechanisms of SAL in the treatment of RA.

Major Biological Effects	Mechanism	In Vivo/Vitro Models
**Improve exercise endurance**	Inhibit the production of O_2_(-)* in blood and liver. Increase the plasma concentrations of malondialdehyde. Upward adjustment of the expressions of Mn-superoxide dismutase, Cu/Zn-superoxide dismutase, and catalase	Acute intake of *Rhodiola rosea* experiment Swimming-induced oxidative stress experiment in rats
**Energy metabolism effects**		
(1) Glycogen storage effects	Enhance endurance and fight fatigue (mitochondrial function) Inhibit foam cell formation (anti-atherosclerosis)	Rat weight-bearing experiment Macrophage induction program
(2) Promote lipid metabolism	Enhances mitochondrial metabolism, iron metabolism, lipid metabolism, and redox metabolism in the brains Inhibit the generation of ROS and lipid accumulation through PI3K/AKT/Gsk3-β Activate AMPK signal transduction Galactose metabolism and fatty acid metabolism	Fatty liver model in laying hens Lipid accumulation model of primary hepatocytes
(3) Repair mitochondrial dysfunction	Anti-polarization of M1 macrophages and protection of mitochondrial function Inhibit the growth, proliferation, migration and metabolism of melanoma	Macrophage cell lines of mice or humans Human melanoma cell lines
**Regulate amino acid metabolism**	CREB/MITF/tyrosinase pathway Bip, CHOP, *p*-PERK, *p*-IRE1α	CIA mouse model Cytotoxicity model of HUVECs induced by hcy
**Reduce metabolite accumulation**	Regulating homeostasis dysregulation Reduce oxidative stress damage Reduce oxidative stress, reduce MDA, H_2_O_2_ and lactate levels in liver and muscle tissue	CIA Model Mouse exhaustion experiment
**Antioxidant activity**	Directly bind to and inhibit HSP90 Target and inhibit the iNOS/COX-2 and NF-κB pathways, and activate the PI3K/Akt/mTOR pathway Reduce infarct area, inhibit apoptosis (cleaved caspase-3, Bax/Bcl-2), lower MDA, and upregulate SOD, CAT, Nrf2, and Trx1. Activate the IGF1R and *p*-ERK1/2 signaling pathways	Lps-induced injury model H9C2 cardiomyocytes MCAO rat model (cerebral artery occlusion) PC12 cells (OGD/R model) Testicular ischemia-reperfusion model
**Regulate immune system**	Inhibit the PI3K/AKT signaling pathway Regulate the HDAC1-HSP90AA1-NFKB2-IKBK-TNF-α axis IL-17/nf-kappa B GSK-3β/nrf2 STAT3/HIF-1α/RORγt pathway Regulate the cell cycle and the expression of downstream genes	CIA rat model TNF-α -induced HFLS-RA model Mouse model of colitis Hepatocyte death and apoptosis model
**Interfere with the synthesis and release of neurotransmitters in the brain**	Increase the expression of BDNF/TrkB and reduce the levels of NE and 5-HT Inhibit NADPH oxidase-mediated oxidative stress, suppress NF-κB activation, and downregulate inflammatory factors such as COX-2 and iNOS Reduce apoptosis and mitochondrial membrane potential collapse, and inhibit the accumulation of NO, nNOS, iNOS, ROS and intracellular Ca^2+^. Activating the Nrf2/ARE pathway for antioxidant effects, inhibiting the P2X7/NF-κB/NLRP3 axis for anti-inflammatory effects, reducing IL-6 and TNF-α, enhancing monoaminergic neurotransmission (5-HT, NE, DA), and stimulating hippocampal neurogenesis (through the SIRT1/PGC-1α pathway). Activate the Nrf2/ARE pathway as an antioxidant and inhibit the P2X7/NF-κB/NLRP3 axis Inhibit the activity of caspase-3 and antagonize the production of NO and NOS.	Lps-induced inflammation model Aβ_1-40_-induced rat model A cognitive impairment model induced by isoflurane MPP^+^-induced PC12 cell injury model A rat tic model induced by IDPN Chronic stress model

## Abbreviations

5-HT: 5-hydroxyserotonin; DA, dopamine; ATP, adenosine triphosphate; ROS, reactive oxygen species; RNS, reactive nitrogen species; TNF-α, tumor necrosis factor-alpha; IL-1β, interleukin-1; IL-6, interleukin-6; HPA axis, hypothalamic–pituitary–adrenal axis; NF-κB, nuclear factor kappa B; MMP, matrix metalloproteinase; AD, Alzheimer’s disease; SAL, salidroside; SOD, superoxide dismutase; NASH, non-alcoholic steatohepatitis; OP, osteoporosis; Hcy, homocysteine; POF, premature ovarian failure; BBB, blood–brain barrier; TGF-β1, transforming growth factor-β1; IDO, Indoleamine 2,3-dioxygenase; AA, acquired aplastic anemia; DMARD, disease-modifying anti-rheumatic drugs; tsDMARD, including methotrexate, targeted synthetic; SF, synovial fibroblast; SM, synovial macrophage; Th1/2, type 1/2 helper T cell; ICOS, inducible T cell co-stimulators CIA, collagen-induced arthritis; VNS, vagus nerve stimulation; CVD, cardiovascular disease; HRVB, heart rate variability; RBC, red blood cells; ACD, anemia of chronic disease; NSAID, non-steroidal anti-inflammatory drugs; ATD, autoimmune thyroid disease; Aβ, beta-amyloid; UGT, uridine diphosphate glycosyltransferase; DHEA, dehydroepiandrosterone; DHEAS, dehydroepiandrosterone sulfate; AA, androgen; SLE, systemic lupus erythematosus; CAP, cholinergic anti-inflammatory pathway; TCM, traditional Chinese medicine; LPO, lipid peroxidation; HUVECs, human umbilical vein endothelial cells; ER, endoplasmic reticulum; Bip, binding immunoglobulin protein; CHOP, and C/EBP homologous protein; PERK, phosphorylation of extracellular regulated protein kinases; IRE1α inositol-requiring enzyme 1α; Tph, peripheral helper T; MDA, malondialdehyde; H_2_O_2_, hydrogen peroxide; GSH, glutathione; BMDM, bone marrow-derived macrophage; DSS, dextran sulfate sodium; EMT, epithelial–mesenchymal transition; t-BHP, tert-butyl hydrogen peroxide; GPX4, glutathione peroxidase 4; α-SMA, α-smooth muscle actin.
